# Approaches and progress in breeding drought‐tolerant maize hybrids for tropical lowlands in west and central Africa

**DOI:** 10.1002/tpg2.20437

**Published:** 2024-02-20

**Authors:** Abebe Menkir, Ibnou Dieng, Melaku Gedil, Wende Mengesha, Muhyideen Oyekunle, Priscillia F. Riberio, Gloria Boakyewaa Adu, Abdoul‐Madjidou Yacoubou, Mmadou Coulibaly, Folusho A. Bankole, John Derera, Bunmi Bossey, Nnanna Unachukwu, Yinka Ilesanmi, Silvestro Meseka

**Affiliations:** ^1^ International Institute of Tropical Agriculture (IITA) Ibadan Nigeria; ^2^ Institute for Agricultural Research/Ahmadu Bello University Zaria Nigeria; ^3^ Crop Research Institute Kumasi Ghana; ^4^ Savanna Agricultural Research Institute Tamale Ghana; ^5^ National Institute of Agricultural Research of Benin/CRA Cotonou Benin Republic; ^6^ Institute de Economie Rurale du Mali Bamako Mali; ^7^ Plant Science Department University of Ilorin Ilorin Nigeria

## Abstract

Drought represents a significant production challenge to maize farmers in West and Central Africa, causing substantial economic losses. Breeders at the International Institute of Tropical Agriculture have therefore been developing drought‐tolerant maize varieties to attain high grain yields in rainfed maize production zones. The present review provides a historical overview of the approaches used and progress made in developing drought‐tolerant hybrids over the years. Breeders made a shift from a wide area testing approach, to the use of managed screening sites, to precisely control the intensity, and timing of drought stress for developing drought‐tolerant maize varieties. These sites coupled with the use of molecular markers allowed choosing suitable donors with drought‐adaptive alleles for integration into existing elite maize lines to generate new drought‐tolerant inbred lines. These elite maize inbred lines have then been used to develop hybrids with enhanced tolerance to drought. Genetic gains estimates were made using performance data of drought‐tolerant maize hybrids evaluated in regional trials for 11 years under managed drought stress, well‐watered conditions, and across diverse rainfed environments. The results found significant linear annual yield gains of 32.72 kg ha^−1^ under managed drought stress, 38.29 kg ha^−1^ under well‐watered conditions, and 66.57 kg ha^−1^ across multiple rainfed field environments. Promising hybrids that deliver high grain yields were also identified for areas affected by drought and variable rainfed growing conditions. The significant genetic correlations found among the three growing conditions highlight the potential to exploit the available genetic resources and modern tools to further enhance tolerance to drought in hybrids.

## Core Ideas

- Drought affects the growth and development of maize in smallholder farmers’ fields.
- Phenotyping sites providing precise control of the severity of drought stress permitted the selection of suitable drought‐tolerant parents for developing maize hybrids.
- Genetic gain estimates from 11 years of data on maize hybrids found significant yield gains under managed drought stress, well‐watered conditions and across multiple rainfed environments.

**Abbreviations**
AMS: African maize stress
CIMMYT: International Maize and Wheat Improvement Center
COMH: commercial hybrids
DTH: drought‐tolerant hybrids
Ex‐PVP: expired plant variety protection certificates
FLV: farmers preferred local variety
GEBV: genomic estimated breeding values
IITA: International Institute of Tropical Agriculture
MARS: marker‐assisted recurrent selection
MDS: managed drought stress
MET: multiple rainfed test environments
MSV: maize streak virus disease
NARS: National Agricultural Research Systems
OPVs: open‐pollinated maize varieties
RCGS: rapid cycle genomic selection
SNP: single nucleotide polymorphism
SSA: sub‐Saharan Africa
WCA: West and Central Africa
WW: well‐watered conditions

## 1 BACKGROUND

Maize exhibits remarkable adaptability to diverse agroecological zones in sub‐Saharan Africa (SSA), with excellent prospects to make significant contributions to bridge the gap between food supply and demand in the continent. It provides more than 30% of the dietary energy and 8%–10% protein to over 300 million consumers (Nuss & Tanumihardjo, 2010), and its cultivation has recorded an annual area growth of 1.69% from 1961 to 2021 (FAOSTAT, 2023). Although this growth in the cultivated area was accompanied by a 2.75% annual increase in total grain production, the observed yearly yield increase of 1.10% is low to meet the growing demands for fast‐growing populations in SSA (FAOSTAT, 2023). Drought contributes more to the low growth rate in grain yields than low soil fertility, weeds, pests, diseases, and limited access to inputs and improved seeds (Cairns, Hellin, et al., 2013). Over the period of 1991–2008 alone, drought affected more than 100 million people in SSA (Shiferaw et al., 2011). Tesfaye et al. (2015) stated that the largest reduction in maize grain yields from drought would occur in Southern and Western Africa by 2050.

Studies show that drought has become more frequent, severe, and widespread in SSA during the last 50 years (Mashih et al., 2014; Mulungu & Ng'ombe, 2019). Climate change‐induced rising temperatures and increasing rainfall variations in SSA will further accentuate the intensity and frequency of drought, leading to more pronounced reductions in maize grain yields under rainfed conditions (Mulungu & Ng'ombe, 2019). Drought stress coinciding with flowering and grain‐filling stages of maize reduces yield by 21%–90% (denmead & shaw, 1960; neSmith & ritchie, 1992; Sah et al., 2020). Millions of farmers living in drought‐affected areas could thus benefit from maize varieties that deliver higher grain yields under drought stress and favorable growing conditions to produce sufficient food for their families and urban consumers. Further increases in yield and yield stability of maize cultivars in farmers’ fields that experience frequent droughts are essential to reduce the risk associated with planting under rainfed conditions and may promote the use of fertilizer, other inputs, and improved management practices. High‐yielding and drought‐tolerant maize varieties may also stimulate freeing up land and labor for growing high‐value and nutritious crops in farming communities.

Before 1997, intermediate maturing and stress‐tolerant maize varieties were selected at the International Institute of Tropical Agriculture (IITA) through evaluations of maize germplasm at test locations that experienced periodic drought stress, followed by extensive testing of elite materials across a wide range of environments. The heterogeneous nature of the intensity and timing of drought stress encountered in these test environments contributed to poor selection efficiency and limited progress in drought tolerance. Despite these challenges, many intermediate and late‐maturing maize varieties and hybrids with high yield potential and resistance to the major tropical diseases adapted to lowland savannas were developed. Nonetheless, continued investment and refined breeding approaches for drought tolerance were still needed to sustain maize productivity growth and food security in West and Central Africa (WCA).

A more focused breeding approach to develop maize germplasm with greater levels of tolerance to drought was initiated in 1997 through a International Maize and Wheat Improvement Center (CIMMYT)‐IITA collaborative project known as the African maize stress (AMS) Project that was implemented across three regions in SSA for 5 years. As breeding for drought tolerance hinges on screening maize germplasm at low rainfall sites for successful induction of a carefully controlled water deficit at flowering and grain‐filling stages of the maize crop (Bänziger et al., 2000), the AMS project supported the establishment of screening facilities at IITA and two National Agricultural Research Systems (NARS) breeding sites in Burkina Faso and Senegal. These facilities were used to spearhead the screening of thousands of families, lines, and open‐pollinated maize varieties (OPVs) from IITA, and the NARS breeding programs, leading to the selection of drought‐tolerant OPVs for direct release or inbred lines and improved germplasm as parents in maize breeding programs in WCA.

Given that the response to drought is multigenic with complex signaling pathways (Mendes et al., 2023), further breeding investment was required to develop new stress‐resilient maize varieties with smallholder farmer‐ and consumer‐preferred traits adapted to diverse production zones. Building on the long‐term achievements of the AMS project, the drought‐tolerant maize for Africa, the stress‐tolerant maize for Africa, and the accelerated genetic gain projects have been sequentially implemented by CIMMYT and IITA for many years across the major maize‐producing countries in Eastern, Southern, and Western Africa. These projects promoted the use of conventional and cutting‐edge molecular breeding tools to develop more productive and stress‐tolerant OPVs and hybrids and increase deployment primarily through the engagement of private seed companies. These projects catalyzed the registration, release, and commercialization of many drought‐tolerant maize varieties and hybrids with desirable adaptive and agronomic attributes in WCA.

In this review, we will provide a historical overview of the approaches used at IITA to mine favorably alleles from diverse adapted and exotic source germplasm and harness conventional and new tools to develop and deploy drought‐tolerant maize hybrids adapted to tropical lowlands in WCA. To highlight the progress made in breeding for drought tolerance and establish benchmarks for further support in breeding intermediate maturing tropical hybrids, we used historical data from collaborative regional trials conducted by national and private sector partners to estimate genetic gains in grain yield.

**Core Ideas**

- Drought affects the growth and development of maize in smallholder farmers’ fields.
- Phenotyping sites providing precise control of the severity of drought stress permitted the selection of suitable drought‐tolerant parents for developing maize hybrids.
- Genetic gain estimates from 11 years of data on maize hybrids found significant yield gains under managed drought stress, well‐watered conditions and across multiple rainfed environments.

## 2 SYSTEMATIC IDENTIFICATION AND EXPLOITATION OF DROUGHT TOLERANCE DONOR GERMPLASM

**FIGURE 1:**
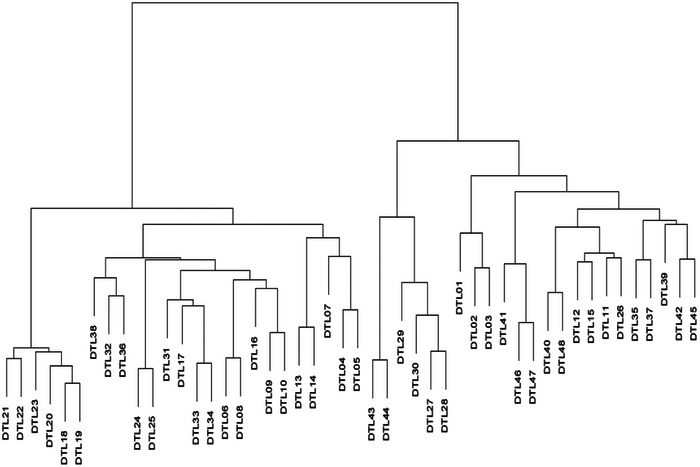
Classifying 48 first‐cycle drought‐tolerant lines using hierarchical cluster analyses with amplified fragment length polymorphism markers.

Access to and use of adapted and introduced drought‐tolerant maize germplasm is a cornerstone for improving yield potential and stability under drought stress. Prior to 1997, IITA developed elite OPVs, inbred lines, and hybrids from diverse source germplasm through wide area testing in the tropical lowlands of WCA. The first search for drought tolerance under the AMS project had thus focused on screening the adapted genetic materials across established drought screening sites at IITA and the NARS. This approach led to the selection of the first generation of drought‐tolerant OPVs for fast‐tracking release and elite parental lines to generate source populations of new parental lines of hybrids for further testing. Additional drought‐tolerant donor source populations from CIMMYT (DTP1W C7, Tuxepeno Sequia C8, and Laposta Sequia C4) and landraces collected from drought‐affected areas of Nigeria and Burkina Faso (Menkir & Akintunde, 2001; Menkir et al., 2009) were screened under carefully managed drought stress (MDS) to identify promising accessions for broadening the genetic base of the adapted drought‐tolerant maize germplasm. The introduced donor populations and landraces had therefore been sources of the first‐cycle inbred lines for use as parents to pre‐package drought tolerance alleles into adapted genetic backgrounds to boost the productivity of maize in drought‐affected areas in WCA. To facilitate utilization of the first‐cycle maize inbred lines to develop new inbred lines with greater tolerance to drought, seven promising first‐cycle donors and 41 elite lines were genotyped with 16 amplified fragment length polymorphism primer pairs. These lines were separated into two major groups (G‐I and G‐II), explaining 21% of the total genetic variation among lines (Figure 1). The remaining 79% of genetic differences among lines resided within groups, reflecting the high genetic diversity available to develop new drought‐tolerant lines. The first sub‐group in G‐I contained lines derived from broad‐based populations (TZL COMP3 and DTPL‐W‐C7), while the second sub‐group includes lines derived mainly from bi‐parental crosses and backcrosses of TZi3 (Table S1). In G‐II, the first sub‐group consists of lines having Babangoyo either as a recipient or a donor parent in backcrosses involving temperate germplasm (MO17LPA and GT‐MAS; Gk) as donors of desirable agronomic traits. The second sub‐group in G‐II consists of lines derived from populations (LATANTE, POP43‐SRC9, Obatanpa, and ACR‐86) and backcrosses having temperate lines as donors of desirable traits and adapted lines as recipients. These results facilitated the selection of suitable parents with diverse genetic backgrounds to develop new maize inbred lines while maintaining the existing heterotic patterns in our breeding program established for superior hybrid performance under stressful conditions.

Our breeding program has continued to introduce exotic maize inbred lines to further broaden and diversify the genetic base of adapted drought‐tolerant lines. Maize inbred lines originating from different breeding programs represent desirable sources of novel drought tolerance alleles (Bohnert et al., 2006; Ribaut et al., 2002) that could be combined easily and more rapidly with other desirable traits in genetic backgrounds adapted to the tropical lowlands (Eberhart et al., 1995; Goodman, 1999). A diversity panel of 359 advanced drought‐tolerant maize inbred lines assembled from tropical maize breeding programs worldwide (Cairns, Crossa, et al., 2013; Wen et al., 2011) were then introduced and evaluated for per se performance under MDS at Ikenne in Nigeria in 2008. More than 70 promising inbred lines with desirable agronomic features and diverse genetic backgrounds were selected as parents for crossing with adapted drought‐tolerant lines to develop source populations for new inbred lines. Furthermore, temperate lines with expired plant variety protection certificates (Ex‐PVP) from the Unites States have been recently introduced as donors of alleles for high yield potential, short plant stature, low ear placement, upright leaves, tolerance to lodging, and resistance to diseases and insects to form backcrosses for developing new drought‐tolerant parents of hybrids and synthetics adapted to tropical lowlands in WCA.

Studies were then conducted to determine the heterotic affinities of the selected exotic lines for systematic introgression into adapted lines while maintaining the existing heterotic groups intact to develop productive hybrids. Adebayo et al. (2014) examined the genetic divergence of 24 promising drought‐tolerant CIMMYT maize inbred lines selected from the diversity panel (Wen et al., 2011) and 24 elite drought‐tolerant IITA lines containing temperate germplasm using simple sequence repeat markers. Cluster analyses showed a clear genetic divergence between the two sets of inbred lines. In a parallel field study, the highest yielding and drought‐tolerant hybrids (DTH) were crossed between CIMMYT and IITA lines (Adebayo et al., 2014), indicating the presence of superior and complimentary novel alleles in the lines originating from the two centers. The selected promising IITA and CIMMYT drought‐tolerant lines were then used for systematic crossing to develop new drought‐tolerant parental lines of superior maize hybrids and source populations of new inbred lines. Maazou et al. (2022) also used both single nucleotide polymorphism (SNP) markers and testcross performance of 24 temperate Ex‐PVP maize inbred lines and two well‐characterized tropical inbred testers representing two heterotic groups in our breeding program. Although yield‐based assessment assigned more than 70% of the Ex‐PVP inbred lines into two groups consistent with existing heterotic patterns, SNPs did not separate the lines along the same line. The resultant yield‐based heterotic affinities of the Ex‐PVP lines, their general combining ability effects for grain yield, and genetic distance from testers have been used as the bases to select superior Ex‐PVP donor parents to develop backcrosses for generating productive and stress‐resilient new parents that can optimize the expression of heterosis in tropical hybrids under both stressful and favorable growing conditions.

## 3 HARNESS PHENOTYPING FACILITIES, CONVENTIONAL, AND NEW BREEDING TOOLS TO ENHANCE DROUGHT TOLERANCE

Robust screening facilities are prerequisites to enhance selection responses and improve genetic gains in grain yield and other traits under drought stress in maize (Edmeades et al., 1999). An existing drought screening site at Ikenne has thus been continually upgraded since 2008 to impose more uniform drought stress at the reproductive stage of the maize crop for more precise and rapid identification of favorable drought tolerance alleles that could be combined with adaptive traits to boost performance in smallholder farmers’ fields. This phenotyping site has permitted the evaluation of increasing numbers of early and advanced generation lines, hybrids, and OPVs for tolerance to drought. It has also been used to unravel and manipulate the underlying genetic basis of drought tolerance and evaluate large numbers of breeding materials for resistance to the major tropical foliar diseases for developing hybrids and OPVs attractive to smallholder farmers and seed companies.

Breeders at IITA employed proven conventional breeding methods, including recurrent selection, backcrossing, and pedigree selection, to exploit native genetic variation for developing drought‐tolerant maize germplasm with other desirable adaptive traits in the last two decades. We screened diverse maize germplasm at Ikenne under carefully controlled moisture deficits coinciding with flowering and grain filling stages of maize and under well‐watered conditions (WW) to identify alleles linked to improved performance for unpredictable rainfed conditions (edmeades et al., 1997). Source populations formed from crosses of selected exotic donor germplasm and adapted populations were then subjected to a selfed progeny selection scheme to continually increase the frequency of favorable drought tolerance alleles under MDS and WW. The best 25%–30% of the lines that combined high grain yields, short anthesis‐silking interval, increased ears per plant, and reduced leaf senescence under MDS with acceptable grain yields under WW had been selected using an index and inter‐mated to form the new selection cycle. The improved selection cycles of these populations had been sources of drought‐tolerant maize inbred lines for use as parents to develop synthetics, hybrids, and source populations of new inbred lines.

Marker‐assisted recurrent selection (MARS) has also been applied to accumulate many quantitative traits loci in bi‐parental crosses using a subset of SNP markers significantly associated with grain yield and other desirable traits recorded under drought stress (Abdumalik et al., 2017; Bankole et al., 2017). This method increases the frequencies of favorable alleles associated with target stress‐resilient traits through a repeated cycle of selection and intermating of progenies carrying selected SNPs associated with better performance under stress conditions. Bankole et al. (2017) employed MARS to improve drought tolerance in a bi‐parental population and reported a yield gain of 7% per cycle under MDS and 1.2% per cycle under WW, with significant changes in the frequencies of favorable and effective alleles at the target SNP loci. Similarly, Abdumalik et al. (2017) found increases in the frequencies of favorable alleles in another bi‐parental population associated with decreases in heterozygosity due to the fixation of the favorable alleles after two cycles of MARS using selected SNP markers.

Despite the effectiveness of MARS in accumulating favorable stress tolerance alleles in the two bi‐parental populations, the approach demands significant financial resources and land for phenotyping more than 200 progenies derived from each of hundreds of bi‐parental crosses generated every year in an active breeding program. Consequently, IITA has recently shifted its focus to rapid cycle genomic selection (RCGS) in two broad‐based populations representing two heterotic groups to improve tolerance to drought and resistance to *Striga hermonthica*. The RCGS used models to predict genomic estimated breeding values (GEBV) based on phenotypic data recorded under MDS, and artificial *Striga* infestation using mid‐density SNPs to select candidate progenies for intermating. Subsequent improvement in these populations has been based on GEBV of SNP markers to select and fix favorable complementary alleles at different loci in the two populations to guarantee the development of divergent and new stress‐tolerant maize inbred lines. Promising progenies selected from each population for intermating to form an advanced selection cycle have also been self‐pollinated to develop new and divergent maize inbred lines. Such unrelated maize inbred lines may form single‐cross hybrids with superior agronomic performance under stressful and favorable conditions through an expression of the dominance effects of genes associated with the heterozygous loci (Falconer & Mackay, 1996; Lamkey & Edwards, 1999). This approach may also facilitate monitoring of the extent of genetic diversity within and between the two populations to ensure long‐term improvement, like the reciprocal recurrent selection program.

## 4 DEVELOPING AND IDENTIFYING SUPERIOR PARENTAL LINES OF HYBRIDS

IITA has extensively used the pedigree approach to cross the best first‐cycle drought‐tolerant maize inbred lines containing CIMMYT and Ex‐PVP germplasm in their genetic backgrounds with elite IITA drought‐tolerant lines to further increase yield potential, tolerance to drought, and other adaptive traits. Early generation lines (S1–S3) have thus been extracted from bi‐parental crosses and backcrosses of drought‐tolerant lines and subjected to repeated inbreeding with phenotypic selection for desirable agronomic traits and resistance to the major foliar disease. One of the major foliar diseases endemic in SSA is the maize streak virus disease (MSV), causing an annual yield loss of US$120 million (Martin & Shepherd, 2009). The early‐generation lines developed with continual introgression of the diverse exotic donor source germplasm have thus been screened for resistance to MSV using three functional SNP markers developed by Nair et al. (2015) and subsequently validated for their usefulness in early‐generation tropical maize lines with diverse genetic backgrounds developed in our breeding program (Sime et al., 2021). These diagnostic SNP markers for MSV resistance have permitted the screening of thousands of S2 lines annually, resulting in significant cost saving during inbreeding to develop promising S3 lines. The selected S3 lines have then been evaluated under MDS during the dry season to select drought‐tolerant lines for advancement to S4 during the main growing season.

**FIGURE 2:**
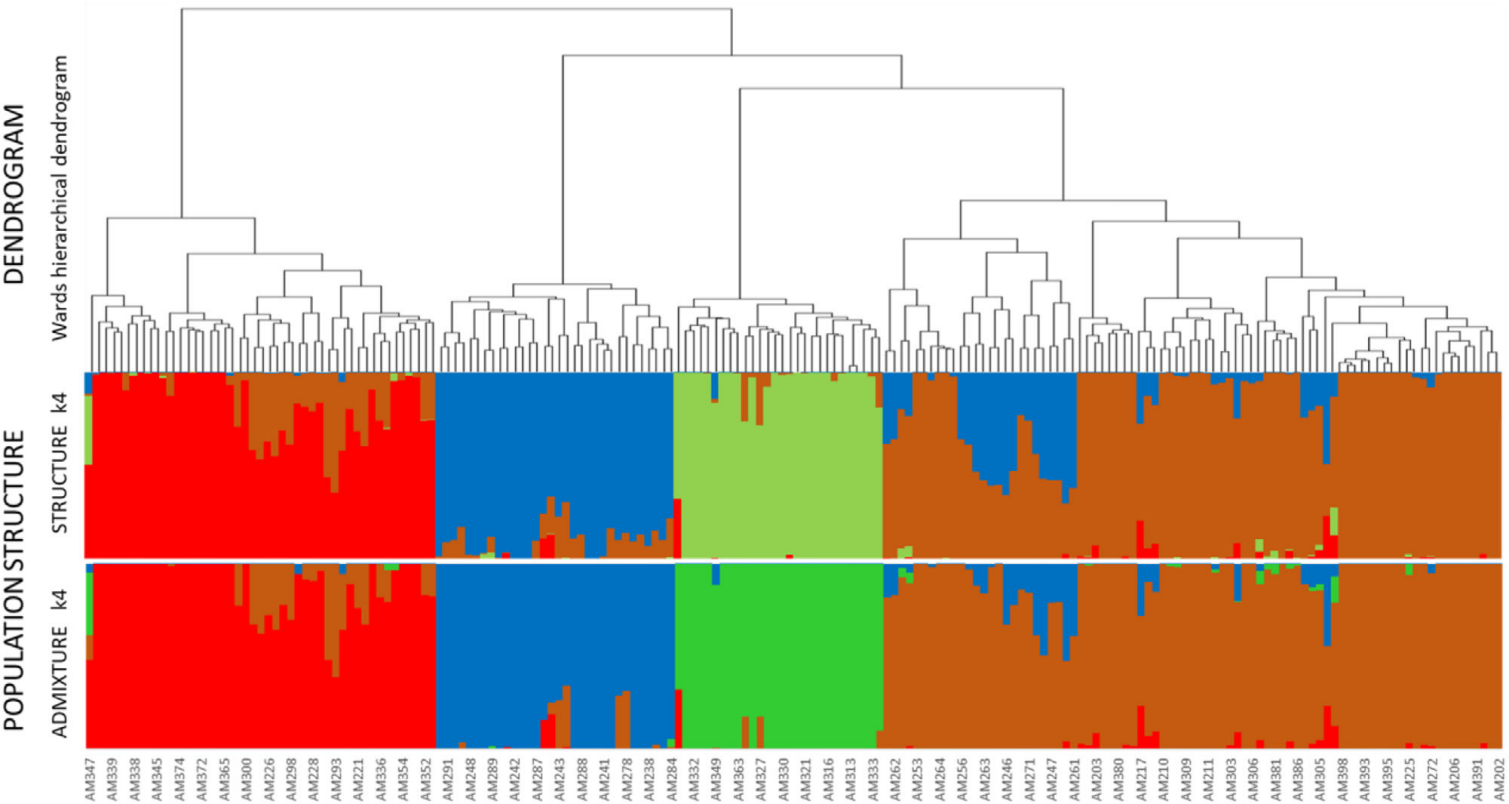
Classifying 190 drought‐tolerant lines using hierarchical cluster, structure, and admixture analyses.

The genetic diversity of 190 advanced drought‐tolerant lines derived from crosses between the first‐cycle drought‐tolerant donor lines and adapted drought‐tolerant inbred lines was assessed using 3633 SNP markers. Cluster, structure, and admixture analyses separated the lines into four major groups, each having sub‐groups, indicating the presence of substantial genetic diversity among these inbred lines (Figure 2). Close to 90% of the lines included in the first and third clusters were derived from crosses between parents containing a common founder inbred line (TZi3) and two first cycle (DT‐SR‐W‐3‐3‐2‐1‐1‐B*9 and P43SRC9FS100‐1‐1‐8‐#1‐B1‐13‐B1‐B*6) and a CIMMYT line (CML373) donor lines. The second cluster comprised 67 yellow and nine white maize inbred lines, with many having KU1414‐SR in their genetic backgrounds. Cluster 4 consisted of 61 white inbred lines derived from 12 crosses of DT lines with diverse genetic backgrounds and 21 yellow inbred lines of diverse origins. The results of this diversity assessment, coupled with pedigree information, were used to assign the lines into two heterotic groups, each of white and yellow breeding pipelines for subsequent development of hybrids and source populations of new inbred lines. Some of these lines have also been shared with the NARS and private sector partners for use as unrelated new parents to develop their own hybrids and as sources of diverse alleles to broaden the genetic base of partners’ tropical stress‐tolerant maize germplasm.

## 5 DEVELOPING DROUGHT‐TOLERANT HYBRIDS FOR TROPICAL LOWLANDS

The development of DTH that are attractive to seed companies for commercialization to smallholder farmers depends on the selection of parental lines combining tolerance to drought with desirable agronomic and adaptive traits (Gedil & Menkir, 2019). In our breeding program, promising drought‐tolerant lines at the S4 and subsequent inbreeding generations have been evaluated in top cross hybrids with at least one inbred or single cross tester belonging to an opposite heterotic group in Stage 1, Stage 2, and Stage 3 trials under MDS during the dry season and across locations during the main growing season. The highest yielding 10%–15% of the DTH with desirable adaptive traits and parental lines with good combining ability are then selected from Stage 1 and Stage 2 trials for further testing in hybrid combinations and for use as parents of new source populations of inbred lines. In Stage 3 trials, the highest yielding 15%–20% of the hybrids involving male and female parents with good synchrony in flowering time, plant height, desirable level of productivity are selected. Their seeds are multiplied for packaging in regional trials to share with the national partners and private seed companies for testing in 11–35 locations. The results of analyses of the regional trial data have been used by partners for making advancement decisions to select the highest yielding and adapted hybrids for further on‐farm testing and eventual registration and release in their respective countries.

## 6 ASSESSING GENETIC GAIN FOR DROUGHT TOLERANCE IN TROPICAL MAIZE HYBRIDS

Estimating genetic gains is vital to determining the effectiveness of the breeding approach used over the years in achieving the required improvements to meet the present and future demands for maize grain. In a dynamic breeding program, new hybrids are constantly added while inferior ones are removed in trials conducted yearly in different locations, leading to unbalanced trial data. Breeding programs striving to continually develop superior DTH with desirable packages of traits routinely analyze such datasets using linear mixed models to obtain reliable genetic gain estimates (de la Vega et al., 2007; Liu et al., 2015; Mackay et al., 2011; Piepho & Möhring, 2006). Using similar data sets, Prasanna et al. (2021) provided a comprehensive summary of significant genetic gains achieved under managed drought, random drought, and favorable growing conditions for CIMMYT early and intermediate breeding pipelines. In the present review, data recorded from drought‐tolerant collaborative regional hybrid trials conducted in WCA have thus been analyzed to determine the impact of the breeding approach used at IITA on developing DTH with superior agronomic performance across stressful and favorable growing conditions (Supporting Information).

**FIGURE 3:**
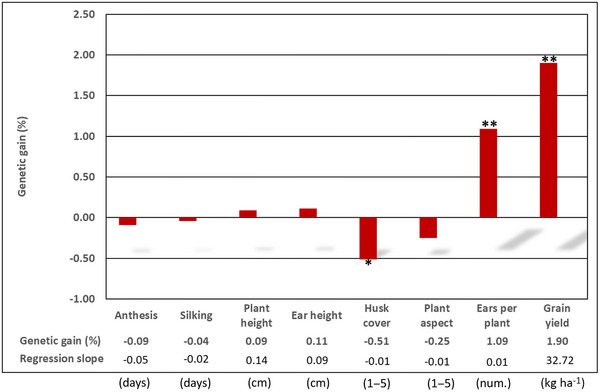
Estimated genetic gains for traits recorded in regional trials evaluated under managed drought stress at Ikenne in Nigeria from 2011 to 2021. Estimates showing * and ** were significantly different at *p* < 0.05 and *p* < 0.01, respectively.

**FIGURE 4:**
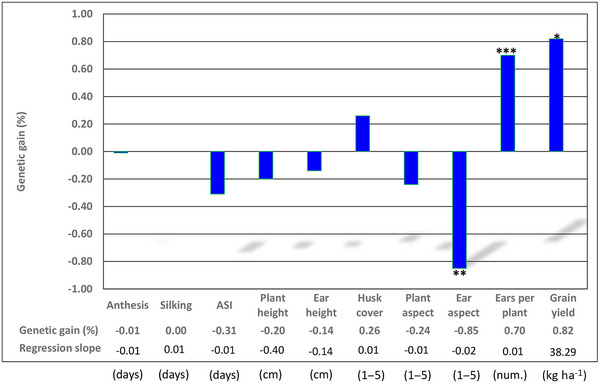
Estimated genetic gains for traits recorded in regional trials evaluated under well‐watered conditions at Ikenne in Nigeria from 2011 to 2021. Estimates showing *, **, and *** were significantly different at *p* < 0.05 and *p* < 0.01, *p* < 0.001, respectively.

The collaborative regional hybrid trials evaluated under MDS were exposed to minimal or no rainfall for 30–33 days prior to anthesis and received no irrigation after that (Figure S1), whereas those grown under WW received irrigation from planting to harvest. The resulting yearly trial mean grain yields varied from 1101 to 3063 kg ha^−1^ under MDS and 3735–6616 kg ha^−1^ under WW. The yearly trial average grain yields under MDS represented 18%–76% of the grain yields under the WW environment, with 10 of the 11 years sustaining yield reductions comparable to the 40%–60% average yield losses considered appropriate for assessing drought tolerance in maize germplasm (Bänziger et al., 2000). The individual regional trials had grain yield repeatability values varying from 0.48 to 0.89 under MDS and 0.65 to 0.92 under WW, indicative of the high data quality recorded in trials conducted for 11 years. The regression analyses showed a significant linear annual yield gain of 32.72 kg ha^−1^ under MDS (Figure S2), representing a yearly genetic gain of 1.90% with a concomitant significant annual increase of 1.09% in ears per plant and desirable change in husk cover (Figure 3). Likewise, a significant annual yield gain of 38.29 kg ha^−1^ representing a yearly genetic gain of 0.82%, was observed under WW (Figure S2), which was associated with significant increases in the number of ears per plant and desirable changes in ear aspect scores (Figure 4).

**FIGURE 5:**
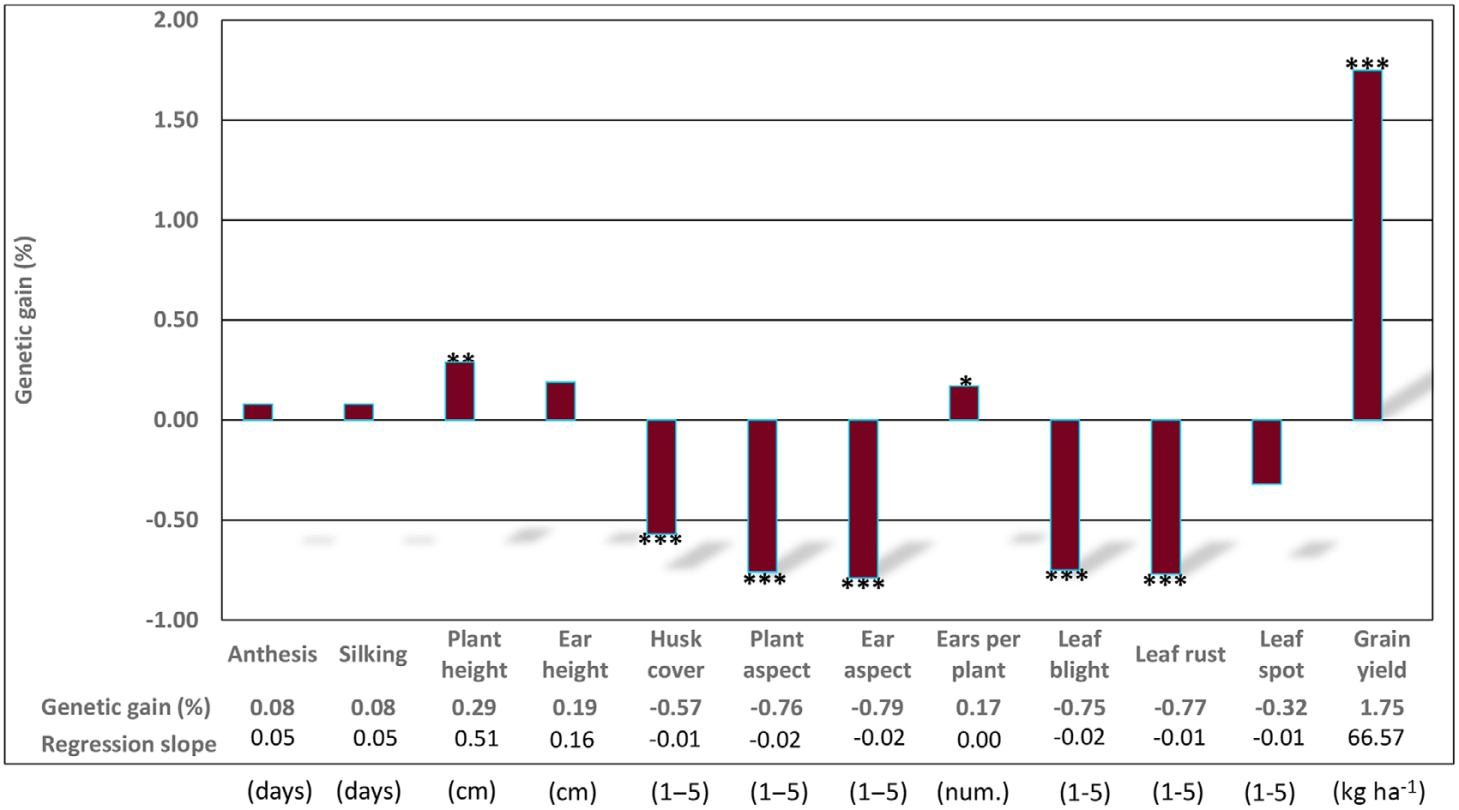
Estimated genetic gains for traits recorded in regional trials evaluated across multiple test environments in West Africa from 2011 to 2021. Estimates showing *, **, and *** were significantly different at *p* < 0.05 and *p* < 0.01, *p <* 0.001, respectively.

The hybrids included in cooperative regional trials were also evaluated in 249 diverse rainfed field environments in WCA to determine whether improvements in grain yield under MDS and WW translate to improvements in agronomic performance in the target population of environments (Figure S3). Among these test environments, 89% exhibited yield repeatability values varying from 0.20 to 0.98 that were used to estimate genetic gain. The average trial mean grain yields of the 221 environments ranged from 833 to 8766 kg ha^−1^, with nearly 25% having trial means of less than 3000 kg ha^−1^. The regression analyses (Figure S2) showed a significant annual yield increase of 66.57 kg ha^−1^, representing a relative genetic gain of 1.75% year^−1^ (Figure 5). This improvement in grain yield was associated with significant annual increases in plant height (0.29%) and ears per plant (0.17%) and desirable significant changes in husk cover, plant aspect and ear aspect scores, and resistance to southern leaf blight and leaf rust. The changes in anthesis and silking days, ear height, and *Curvularia* leaf spot were not significant (Figure 5). The genetic correlations of grain yield recorded under MDS with that obtained under WW (rg = 0.56 ± 0.05) and across multiple rainfed test environments (MET) (rg = 0.53 ± 0.05) were significant. Similarly, the genetic correlation between grain yields measured under WW and across MET (rg = 0.66 ± 0.05) was significant.

**FIGURE 6:**
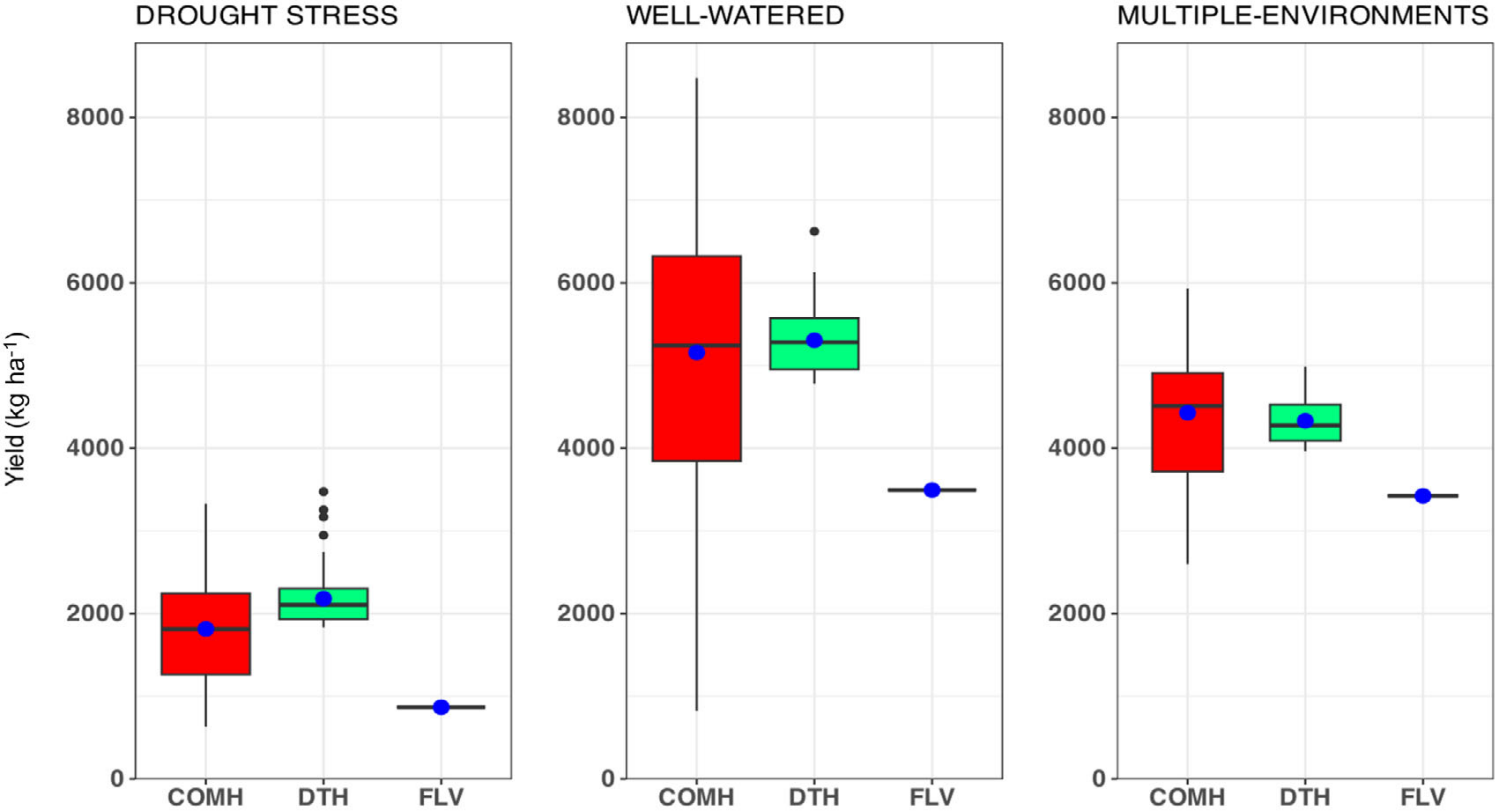
Box plot of average grain yield of drought‐tolerant hybrids (DTH), commercial hybrids (COMH), and farmer's preferred local variety (FLV) evaluated under managed drought stress, well‐watered condition, and across diverse rainfed field environments.

Assessing the potential value of developing hybrids under MDS that also express superior agronomic performance across a broad range of maize‐growing environments is important for commercialization and cultivation in smallholder farmers’ fields. In the present review, we compared the average grain yield of the top 25% of the DTH relative to the average of all the commercial hybrids (COMH) checks representing the best available cultivars from different seed companies. On average, the DTH had a higher grain yield than the COMH and farmers preferred local variety (FLV) under MDS and WW but was competitive to COMH across MET (Figure 6). The DTH exhibited less variation in mean grain yields than the COMH under MDS, WW, and across MET, indicating the potential to identify DTH with consistently high grain yields. Amongst the 148 DTH, 22% produced more than 2000 kg ha^−1^ under MDS and 4000 kg ha^−1^ under WW and across MET (Table S2). On average, the best 12% of the hybrids tested for at least 2 years produced 26% more grain yield than the COMH and 160% more grain yield than the FLV under MDS (Table S2). These hybrids were competitive to the COMH under WW and across MET but outyielded the FLV by 50% under WW and 31% across MET.

## 7 SYNTHESIS OF APPROACHES AND PROGRESS IN BREEDING FOR DROUGHT TOLERANCE

Breeding maize for drought tolerance has been regarded as a viable strategy for decades to minimize yield losses in farmers’ fields. Before the mid‐1990s, stress‐tolerant open‐pollinated maize varieties and hybrids adapted to heterogeneous target production environments in WCA were developed through extensive testing under rainfed conditions experiencing intermittent drought. Limited progress had been made in generating DTH using this approach because of the unpredictable and variable nature of drought occurrences in maize production fields. To achieve significant progress in drought tolerance, managed stress screening sites were established in the late 1990s that allowed the induction of severe moisture deficits before anthesis and during silking and grain‐filling periods of maize. Although greater emphasis had been placed on selecting promising inbred lines and hybrids based on performance under MDS, advanced inbred lines and hybrids had also been selected under WW to ensure that they performed well during years with favorable growing conditions. Careful screening of introduced tropical and temperate maize inbred lines, tropical populations, and landrace collections as sources of drought‐adaptive alleles and other desirable agronomic attributes under MDS led to the development of the first‐cycle drought‐tolerant maize inbred lines. Continual integration of novel alleles derived from the first‐cycle lines into existing adapted elite genetic materials resulted in the development of new maize inbred lines with better tolerance to drought and other adaptive traits. An important observation made during the introgression of novel alleles from introduced maize germplasm into elite maize inbred lines was that the disruption of established heterotic patterns rendered selecting desirable parents difficult for developing new inbred lines and hybrids. Molecular markers and field trials have then been used to determine the heterotic affinities of exotic germplasm to existing heterotic groups for subsequent systematic use in inbred line development. This approach has been effective in selecting parents with desirable attributes within a heterotic group to develop new inbred lines with novel drought‐adaptive alleles and other defensive traits. These lines have been exploited by breeders in IITA and national and private sector partners to generate productive hybrids and better inbred lines with higher levels of tolerance to drought stress.

Maize inbred lines containing exotic source germplasm in their genome developed over the years under MDS were also assayed using molecular markers and field trials to assign them into well‐defined heterotic groups. This approach has been effective in selecting divergent parents imparting superior agronomic performance in hybrids under MDS and WW, consistent with the findings reported in other studies (Bertran et al., 2003; Duvick, 2005; Monneveux et al., 2008). The most promising maize hybrids have also been supplied to the national and private sector partners for extensive regional testing to facilitate the selection of drought‐tolerant adapted maize hybrids for release and commercialization in WCA. Furthermore, the adapted drought‐tolerant maize inbred lines with diverse genetic backgrounds can provide the germplasm base to further incorporate novel alleles and trait combinations to enhance the rate of genetic gain and delivery of higher yields under stressful and favorable growing conditions. These lines can also serve as breeding stock for the national maize breeding programs and seed companies to further improve local adaptation in their countries.

The collaborative regional trials involving intermediate/late‐maturing hybrids conducted by partners for 11 years were also used to examine the effectiveness of the breeding approach in achieving genetic gains under MDS,WW, and across MET. Drought‐induced reductions in mean grain yield observed in 10 of the 11 years fell within the range of losses considered appropriate to elicit differential responses of the hybrids to water deficit (Bänziger et al., 2000; Heisey & Eadmeades, 1999). In addition, the moderate to high grain yield repeatability values estimated under MDS and WW signify that the management of the regional trials was effective in triggering consistent response patterns of the hybrids across the different years. Likewise, nearly 90% of the MET had acceptable to high repeatability values for grain yield, suggesting that the observed differences among hybrids were largely determined by their genetic backgrounds rather than by the differences in weather conditions, soil properties, crop management practices, disease, and pest pressure encountered during the evaluation of the regional trials. Significant annual yield gains were recorded for the three growing conditions, highlighting that selection for drought tolerance facilitated simultaneous improvements in yield gains in controlled, favorable, and variable field environments, consistent with the findings in other studies (Byrne et al., 1995; Edmeades et al., 1999; Masuka et al., 2017). The significant and positive genetic correlations among the three growing conditions also confirm these findings, possibly because the same set of genes elicited similar response patterns of the hybrids across the different growing conditions (Lorenzana & Bernardo, 2008). Therefore, the genetic improvements in drought tolerance achieved in parents imparted constitutive changes in hybrids, leading to the observed yield gains under MDS, WW, and across MET.

The significant genetic correlations also highlight the possibility of selecting high‐yielding hybrids under MDS with high yield potential under WW and across MET. The identification of hybrids producing high grain yields under MDS, WW, and across MET shows the effectiveness of our breeding approach in accumulating desirable traits and allelic combinations in hybrids imparting superior agronomic performance across varying growing conditions. Ghahfarokhi et al. (2016) reported that drought‐tolerant maize hybrids had higher levels of antioxidant activities at vegetative and reproductive stages and produced high grain yields under drought stress. Likewise, Anjum et al. (2016) found a drought‐tolerant maize cultivar expressing higher photosynthetic activity, osmotic accumulation, antioxidant activities, and less peroxidation as opposed to a drought‐susceptible cultivar. It is, therefore, likely that selection for drought tolerance may enhance the accumulation of common favorable molecular, biochemical, and physiological mechanisms that confer broad‐spectrum agronomic performance across stressful and variable production conditions occurring in target environments (Ramegowda & Snthil‐Kumar, 2015; Sun et al., 2015).

## 8 CONCLUSION AND PROSPECTS

The shift from a wide‐area testing approach to establishing managed screening sites allowed reliable evaluation of adapted and introduced maize germplasm at the desired stress intensity and duration, leading to the selection of suitable drought‐tolerant donors with diverse genetic backgrounds. The use of suitable donors of drought‐adaptive alleles together with molecular and conventional breeding tools facilitated the development of maize hybrids with significant yield gains under MDS, WW, and across diverse MET. Promising hybrids were also identified that can deliver high grain yields to areas affected by drought and variable rainfed growing environments. The drought‐tolerant elite inbred lines created over the years may also be used to develop multiple stress‐resilient maize varieties to respond to projected adverse effects of climate variability and change that accentuate the simultaneous presence of drought, heat, and biotic stresses (Pandey et al., 2017; Tesfaye et al., 2015). One strategy that could be pursued to combine tolerance to multiple stresses involves the formation of broad‐based populations by inter‐crossing diverse elite maize inbred lines selected within a heterotic group that is tolerant to different target stresses. Perhaps subsequent improvement of the resulting complementary populations using RCGS to promote continual recombination of favorable stress‐adaptive alleles and increase their frequencies, which is like reciprocal recurrent selection, may offer better management of genetic diversity to accelerate the rate of genetic gain for multiple stress tolerance in hybrids adapted to areas affected by unpredictable co‐occurrence of abiotic and biotic stresses. Furthermore, the drought‐tolerant elite maize inbred lines may serve as invaluable genetic resources for unraveling the underlying physiological, biochemical, and molecular mechanisms of tolerance to use novel tools for strategic pyramiding of factors conditioning multiple stress tolerance in maize.

## AUTHOR CONTRIBUTIONS

**Abebe Menkir**: Conceptualization; investigation; writing—original draft; writing—review and editing. **Ibnou Dieng**: Formal analysis; methodology; writing—review and editing. **Melaku Gedil**: Data curation; investigation; supervision. **Wende Mengesha**: Data curation; methodology. **Muhyideen Oyekunle**: Data curation; investigation. **Priscilla F. Ribeiro**: Data curation; investigation. **Gloria Boakyewaa Adu**: Data curation; investigation. **Abdoul‐Madjidou Yacoubou**: Data curation; investigation. **Mmadou Coulibaly**: Data curation; investigation. **Folusho A. Bankole**: Data curation; investigation. **John Derera**: Writing—review and editing. **Bunmi Bossey**: Data curation; formal analysis. **Nnanna Unachukwu**: Formal analysis; methodology. **Yinka Ilesanmi**: Formal analysis; methodology. **Silvestro Meseka**: Data curation; methodology; writing—review and editing.

## CONFLICT OF INTEREST STATEMENT

The authors declare no conflicts of interest.

## Supporting information

Supplementary Figure S1. Rainfall received during the evaluation of regional trials under stress conditions for 11 years.
Supplementary Figure S2. Genetic garin estimates for grain yield from regional cooperative hybrid trials evaluated under managed drought stress (MDS), well‐watered conditions (WW), and across multiple rainfed field environments (MET).
Supplementary Figure S3. Distribution of test location used for evaluating regional trials for 11 years.

Supplementary Table S1. List of 48 first cycle maize inbred lines selected for tolerance to drought and genotyped with 16 AFLP primers.
Supplementary Table S2. Lis of hybrids included in regional trials and their means recorded under MDS, WW, and across diverse MTE for 11 years.

Supporting Information

## Data Availability

Data generated in this study will be available on the IITA website.
