# Supplementary material for: Approaches and progress in breeding drought‐tolerant maize hybrids for tropical lowlands in west and central Africa
Source: Plant Genome. 2024 Feb 20;17(2):e20437. doi: 10.1002/tpg2.20437 (PMC12806867; doi:10.1002/tpg2.20437)
Supplement: Supplementary file 1 [file TPG2-17-e20437-s001.docx]

Supplementary Figure S1. Rainfall received during evaluation of regional trials under stress conditions for 11 years.

Supplementary Figure S2. Genetic garin estimates for grain yield from regional cooperative hybrid trials evaluated under manged drought stress (MDS), well-watered (WW) condtion and across multiple rainfed field environments (MET).

Supplementary Figure S3. Distribution of test location used for evaluating regional trials for 11 years.
