# Supplementary material for: Approaches and progress in breeding drought‐tolerant maize hybrids for tropical lowlands in west and central Africa
Source: Plant Genome. 2024 Feb 20;17(2):e20437. doi: 10.1002/tpg2.20437 (PMC12806867; doi:10.1002/tpg2.20437)
Supplement: Supplementary file 2 [file TPG2-17-e20437-s002.docx]

Supplementary Table S1. List of 48 first cycle maize inbred lines selected for tolerance to drought and genotyped with 16 AFLP primers.

| Lines | Cluster*Sub-cluster | Pedigree |
| --- | --- | --- |
| DTL18 | 1*1 | TZL-COMP3-C2-S2-34-4-1-2-B*5 |
| DTL19 | 1*1 | DTPL-W-C7-S2-7-1-1-1-1-B-5-B*5 |
| DTL20 | 1*1 | DTPL-W-C7-S2-7-1-1-1-1-B-4-B*5 |
| DTL21 | 1*1 | DTPL-W-C7-S2-7-1-1-1-1-B-3-B*5 |
| DTL22 | 1*1 | DTPL-W-C7-S2-7-1-1-1-1-B-2-B*5 |
| DTL23 | 1*1 | DTPL-W-C7-S2-1-2-1-1-5-B-1-B*5 |
| DTL04 | 1*2 | DT-SR-W-3-3-2-1-1-B*10 |
| DTL05 | 1*2 | TZi3xHIx4269-1xTZi3-7-1-B*9 |
| DTL06 | 1*2 | TZi3xICAL224-1xTZi3-3-1-B*9 |
| DTL07 | 1*2 | TZi3xPAC90038-1xTZi3-3-1-B*9 |
| DTL08 | 1*2 | TZi3xICAL224-1xTZi3-2-2-B*9 |
| DTL09 | 1*2 | TZi3xPAC90038-1xTZi3-6-1-B*9 |
| DTL10 | 1*2 | TZi3xPAC90038-1xTZi3-6-2-B*9 |
| DTL13 | 1*2 | (KU1403xTZi3)-4-4-1-1-B*7 |
| DTL14 | 1*2 | (KU1403xTZi3)-7-2-1-B*7 |
| DTL16 | 1*2 | (KU1403xTZi3)-7-2-1-B*6 |
| DTL24 | 1*2 | TZi3 x B73LPAX TZi3-7-1-1-1-B*7 |
| DTL25 | 1*2 | TZi3 x B73LPAX TZi3-7-1-2-2-B*7 |
| DTL31 | 1*2 | T115 x 9071-2-1-7-B*10 |
| DTL32 | 1*2 | TZi3 x Mi82-23-1-1-3-B*8 |
| DTL33 | 1*2 | TZi3 x Mi82-23-2-1-2-B*8 |
| DTL34 | 1*2 | TZi3 x Mi82-23-2-1-5-B*8 |
| DTL36 | 1*2 | TZi3 x Mi82-27-4-1-1-B*8 |
| DTL38 | 1*2 | TZi3 x GT-MAS-Gk-37-1-1-1-B*7 |
| DTL27 | 2*1 | Babangoyo x MO17LPA x Babangoyo-23-1-3-1-B*7 |
| DTL28 | 2*1 | Babangoyo x MO17LPA x Babangoyo-23-4-3-3-B*7 |
| DTL29 | 2*1 | Babangoyo x MO17LPA x Babangoyo-23-4-3-4-B*7 |
| DTL30 | 2*1 | Babangoyo x MO17LPA x Babangoyo-28-1-2-1-B*7 |
| DTL43 | 2*1 | (GT-MAS:Gk x BABANGOYO x GT-MAS:Gk)-2-1-3-1-B*9 |
| DTL44 | 2*1 | (GT-MAS:Gk x BABANGOYO x GT-MAS:Gk)-2-1-3-2-B*9 |
| DTL01 | 2*2 | LATA-26-1-1-1-B*7 |
| DTL02 | 2*2 | P43SRC9FS100-1-1-8-#1-B1-13-B1-B*7 |
| DTL03 | 2*2 | CML373 -B*4 |
| DTL11 | 2*2 | Obantapa-33-5-1-B*9 |
| DTL12 | 2*2 | (TZMI501xKU1414x501)-1-4-3-1-B*6 |
| DTL15 | 2*2 | 161-B*4 |
| DTL17 | 2*2 | ACR-86-8-1-2-1-1-1-B-1-B*5 |
| DTL26 | 2*2 | TZi3 x B73LPAX TZi3-17-2-2-1-B*7 |
| DTL35 | 2*2 | TZi3 x GT-MAS-Gk-26-3-1-3-B*8 |
| DTL37 | 2*2 | TZi3 x Mi82-28-1-1-2-B*8 |
| DTL39 | 2*2 | GT-MAS:gk x BABANGOYO-1-3-2-B*9 |
| DTL40 | 2*2 | KU1409 x MO17LPA x KU1409-27-3-1-1-B*6 |
| DTL41 | 2*2 | ((4001 x 9848) x 4001)-29-1-4-B-1-B*10 |
| DTL42 | 2*2 | ((KU1414 x 9450) x 9450)-24-2-1-B-B-B-1-B*10 |
| DTL45 | 2*2 | TZi3 x GT-MAS-Gk-10-3-1-2-B*7 |
| DTL46 | 2*2 | 4001 x B73LPA x 4001-6-2-1-4-B*5 |
| DTL47 | 2*2 | 4001 x B73LPA x 4001-6-2-1-4-B*7 |
| DTL48 | 2*2 | TZi3 x B73LPAX TZi3-17-4-1-1-B*7 |

Supplementary Table S2. Lis of hybrids included in regional trials and their means recorded under MDS, WW conditions and across diverse MTE for 11 years.

|  |  | Grain yield under (kg/ha) | | |
| --- | --- | --- | --- | --- |
| ENTRY | Name | MDS | WW conditions | Across MET |
| DTH001 | M0826-1 | 1511 | 4675 | 3996 |
| DTH002 | M0826-2 | 1616 | 4971 | 3760 |
| DTH003 | M0826-3 | 1706 | 5151 | 4045 |
| DTH004 | M0826-4 | 1339 | 4279 | 3610 |
| DTH005 | M1124-18 | 1720 | 4629 | 3876 |
| DTH006 | M1124-15 | 1731 | 4534 | 3793 |
| DTH007 | M1124-16 | 1687 | 4812 | 3863 |
| DTH008 | M1124-17 | 1989 | 5326 | 3907 |
| DTH009 | M1124-23 | 1996 | 4531 | 3848 |
| DTH010 | M1124-24 | 1957 | 4549 | 4088 |
| DTH011 | M1124-22 | 1776 | 5285 | 4007 |
| DTH012 | M1124-29 | 1587 | 4009 | 3500 |
| DTH013 | M1124-26 | 1832 | 4361 | 3805 |
| DTH014 | M1124-27 | 1857 | 4725 | 4061 |
| DTH015 | M1124-31 | 1658 | 4530 | 3633 |
| DTH016 | M1124-28 | 1441 | 3259 | 3411 |
| COMH017 | COMH017 | 834 | 3198 | 2998 |
| COMH018 | COMH018 | 629 | 3354 | 3326 |
| COMH019 | COMH019 | 1358 | 4566 | 3556 |
| COMH020 | COMH020 | 2420 | 6321 | 4691 |
| COMH021 | COMH021 | 1744 | 3539 | 3717 |
| COMH022 | COMH022 | 1688 | 3833 | 3640 |
| COMH023 | COMH023 | 1152 | 3845 | 3334 |
| COMH024 | COMH024 | 1153 | 1743 | 3376 |
| DTH025 | Local check | 867 | 3493 | 3423 |
| DTH026 | M1124-1 | 1339 | 1848 | 4287 |
| DTH027 | M1124-2 | 1198 | 4917 | 3167 |
| DTH028 | M1124-3 | 2645 | 4558 | 4188 |
| DTH029 | M1124-4 | 1264 | 5045 | 4164 |
| DTH030 | M1124-5 | 1458 | 4599 | 3573 |
| DTH031 | M1124-6 | 1864 | 4246 | 4016 |
| DTH032 | M1124-7 | 1788 | 1653 | 4094 |
| DTH033 | M1124-8 | 1851 | 4838 | 2429 |
| DTH034 | M1124-9 | 2363 | 4254 | 3888 |
| DTH035 | M1124-10 | 2649 | 6040 | 3674 |
| DTH036 | M1124-11 | 2051 | 6622 | 3627 |
| DTH037 | M1124-12 | 2746 | 5842 | 4201 |
| DTH038 | M1124-13 | 2269 | 5728 | 4098 |
| DTH039 | M1124-14 | 1602 | 4717 | 4264 |
| DTH040 | M1124-19 | 2240 | 5454 | 3796 |
| DTH041 | M1124-20 | 2947 | 5333 | 3825 |
| DTH042 | M1124-21 | 3474 | 5528 | 4001 |
| DTH043 | M1124-25 | 2205 | 5613 | 4162 |
| DTH044 | M1124-30 | 2128 | 5003 | 3999 |
| DTH045 | M1227-1 | 2177 | 4156 | 3907 |
| DTH046 | M1227-2 | 2092 | 3862 | 3656 |
| DTH047 | M1227-3 | 1696 | 4469 | 3857 |
| DTH048 | M1227-4 | 1725 | 4375 | 4181 |
| DTH049 | M1227-5 | 1686 | 4901 | 3636 |
| DTH050 | M1227-6 | 1600 | 4328 | 4041 |
| DTH051 | M1227-7 | 1692 | 5216 | 4006 |
| DTH052 | M1227-8 | 1983 | 4488 | 3843 |
| DTH053 | M1227-9 | 2050 | 5025 | 3776 |
| DTH054 | M1227-10 | 2228 | 5138 | 4099 |
| DTH055 | M1227-11 | 2128 | 5530 | 4098 |
| DTH056 | M1227-12 | 1853 | 4699 | 3965 |
| DTH057 | M1227-13 | 475 | 3092 | 3576 |
| DTH058 | M1227-14 | 1217 | 3707 | 3983 |
| DTH059 | M1227-15 | 1019 | 4945 | 3827 |
| DTH060 | M1227-16 | 1177 | 4102 | 3796 |
| DTH061 | M1227-17 | 1413 | 3930 | 3801 |
| DTH062 | M1227-18 | 1756 | 4890 | 3967 |
| DTH063 | M1227-19 | 1101 | 5065 | 3891 |
| DTH064 | M1227-20 | 902 | 3713 | 3762 |
| DTH065 | M1227-21 | 1926 | 3688 | 3852 |
| DTH066 | M1327-1 | 1367 | 3366 | 3012 |
| DTH067 | M1327-2 | 1345 | 4285 | 3824 |
| DHH070 | COMH070 | 1239 | 4013 | 3449 |
| DHH071 | COMH071 | 1265 | 4483 | 3848 |
| DHH072 | COMH072 | 707 | 3720 | 3061 |
| DHH073 | COMH073 | 1188 | 2741 | 3717 |
| DHH074 | COMH074 | 1760 | 3638 | 4511 |
| DHH075 | COMH075 | 1608 | 5617 | 4309 |
| DHH076 | COMH076 | 1907 | 5963 | 4452 |
| DHH077 | COMH077 | 2056 | 6294 | 5070 |
| DTH078 | M1304-5 | 1853 | 4160 | 3941 |
| DTH079 | M1304-16 | 2012 | 4941 | 3999 |
| DTH080 | M1304-18 | 2024 | 4869 | 4098 |
| DTH081 | M1304-23 | 1679 | 4991 | 3565 |
| DTH082 | M1306-11 | 2090 | 3978 | 3362 |
| DTH083 | M1306-13 | 1769 | 4753 | 3946 |
| DTH084 | M1309-66 | 1767 | 5097 | 3897 |
| DTH085 | M1309-67 | 2117 | 6027 | 3964 |
| DTH086 | M1314-48 | 2396 | 5403 | 4056 |
| DTH087 | M1314-75 | 2152 | 4795 | 3936 |
| DTH088 | M1314-76 | 1859 | 5581 | 3721 |
| DTH089 | M1315-3 | 1363 | 4115 | 3380 |
| DTH090 | M1315-20 | 1706 | 3911 | 3346 |
| DTH091 | M1315-24 | 1730 | 3777 | 3562 |
| DHH092 | COMH092 | 2819 | 5268 | 4167 |
| DHH093 | COMH093 | 1349 | 5182 | 4733 |
| DTH094 | M1427-1 | 1886 | 4559 | 3360 |
| DTH095 | M1427-2 | 1535 | 3859 | 3880 |
| DTH096 | M1427-3 | 1988 | 5322 | 4171 |
| DTH097 | M1427-4 | 1828 | 4706 | 4102 |
| DTH098 | M1427-5 | 1794 | 5130 | 4044 |
| DTH099 | M1427-6 | 2346 | 5554 | 4512 |
| DTH100 | M1427-7 | 1778 | 4812 | 3592 |
| DTH101 | M1427-8 | 1856 | 4329 | 4029 |
| DTH102 | M1427-9 | 1904 | 5289 | 3934 |
| DTH103 | M1427-10 | 1801 | 4563 | 3943 |
| DTH104 | M1427-11 | 1836 | 3550 | 3524 |
| DTH105 | M1427-12 | 1848 | 5328 | 4372 |
| DTH106 | M1427-13 | 1914 | 4816 | 3834 |
| DTH107 | M1427-14 | 1771 | 4694 | 4201 |
| DTH108 | M1427-15 | 1699 | 4389 | 4130 |
| DTH109 | M1427-16 | 2197 | 4610 | 3898 |
| DTH110 | M1427-17 | 1504 | 3922 | 3696 |
| DTH111 | M1427-18 | 1785 | 4026 | 3991 |
| DTH112 | M1427-19 | 1252 | 4567 | 3963 |
| DTH113 | M1427-20 | 1439 | 4604 | 3926 |
| DTH114 | M1427-21 | 1014 | 2680 | 2837 |
| DTH115 | M1427-22 | 1040 | 2117 | 3081 |
| DTH116 | M1427-23 | 931 | 3293 | 2881 |
| DTH117 | M1427-24 | 918 | 2706 | 2918 |
| DHH118 | COMH118 | 2242 | 6999 | 4500 |
| DHH119 | COMH119 | 2816 | 7140 | 5284 |
| DTH120 | M1527-1 | 2056 | 5592 | 3805 |
| DTH121 | M1527-2 | 1457 | 3872 | 3406 |
| DTH122 | M1527-3 | 1645 | 4777 | 3921 |
| DTH123 | M1527-4 | 1765 | 4257 | 3440 |
| DTH124 | M1527-5 | 1687 | 4413 | 3481 |
| DTH125 | M1527-6 | 1692 | 4463 | 3951 |
| DTH126 | M1527-7 | 1877 | 4895 | 4005 |
| DTH128 | M1627-1 | 1377 | 4826 | 4456 |
| DTH129 | M1627-2 | 1449 | 3109 | 3624 |
| DTH130 | M1627-3 | 1666 | 4009 | 3877 |
| DTH131 | M1627-4 | 2291 | 5167 | 4385 |
| DTH132 | M1627-5 | 2092 | 5602 | 4168 |
| DTH133 | M1627-6 | 1821 | 5401 | 4443 |
| DTH134 | M1627-7 | 2430 | 5818 | 4383 |
| DTH135 | M1627-8 | 2577 | 5276 | 4334 |
| DTH136 | M1627-9 | 1910 | 4437 | 3912 |
| DTH137 | M1627-10 | 2001 | 5416 | 4260 |
| DTH138 | M1627-11 | 1739 | 4628 | 4366 |
| DTH139 | M1627-12 | 1364 | 4401 | 3692 |
| DTH140 | M1627-13 | 1586 | 5727 | 3888 |
| COMH141 | COMH141 | 2088 | 5866 | 4659 |
| COMH142 | COMH142 | 1590 | 5243 | 4120 |
| COMH145 | COMH145 | 2366 | 7366 | 5779 |
| COMH146 | COMH146 | 1665 | 4223 | 4182 |
| COMH147 | COMH147 | 1811 | 5057 | 4717 |
| COMH148 | COMH148 | 2102 | 6194 | 4854 |
| COMH149 | COMH149 | 2845 | 8478 | 5752 |
| COMH150 | COMH150 | 1856 | 6258 | 4973 |
| COMH151 | COMH151 | 2225 | 7267 | 5905 |
| COMH152 | COMH152 | 2364 | 7398 | 5929 |
| DTH153 | A1807-4 | 2366 | 5444 | 4889 |
| DTH154 | A1807-20 | 1932 | 5841 | 4987 |
| DTH155 | A1807-24 | 2378 | 4845 | 4433 |
| DTH156 | A1807-28 | 2492 | 4514 | 4550 |
| DTH157 | A1807-35 | 1854 | 5307 | 4961 |
| DTH158 | A1807-39 | 2254 | 4909 | 4669 |
| DTH159 | A1807-41 | 2304 | 4797 | 4528 |
| DTH160 | A1807-45 | 2372 | 4786 | 4545 |
| DTH161 | A1807-49 | 1640 | 5330 | 4661 |
| COMH162 | COMH162 | 1181 | 3709 | 4869 |
| COMH163 | COMH163 | 2179 | 7206 | 4678 |
| COMH164 | COMH164 | 879 | 822 | 2599 |
| COMH165 | COMH165 | 1566 | 6990 | 4908 |
| DTH166 | A1909-3 | 1784 | 5847 | 4351 |
| DTH167 | A1909-4 | 3254 | 6128 | 4550 |
| DTH168 | A1909-9 | 2034 | 5202 | 4967 |
| DTH169 | A1909-14 | 2166 | 6022 | 4815 |
| DTH170 | A1909-28 | 2164 | 3853 | 4343 |
| DTH171 | A1909-29 | 2328 | 4693 | 4371 |
| DTH172 | A1909-33 | 2013 | 5329 | 4736 |
| DTH173 | A1909-34 | 2256 | 5372 | 4689 |
| DTH174 | A1909-39 | 2256 | 5956 | 4684 |
| COMH175 | COMH175 | 2401 | 6380 | 5513 |
| COMH176 | COMH176 | 2590 | 5466 | 4798 |
| COMH177 | COMH177 | 1935 | 4788 | 3983 |
| COMH178 | COMH178 | 1954 | 5038 | 4542 |
| COMH179 | COMH179 | 3329 | 6405 | 5671 |
| COMH180 | COMH180 | 681 | 5104 | 4792 |
| DTH181 | A2001-1 | 1933 | 5065 | 4397 |
| DTH182 | A2001-2 | 2223 | 3660 | 4429 |
| DTH183 | A2001-3 | 2041 | 5245 | 4514 |
| DTH184 | A2001-4 | 1653 | 4696 | 4777 |
| DTH185 | A2001-5 | 3169 | 5678 | 4244 |
| DTH186 | A2001-6 | 1399 | 5808 | 4849 |
| DTH187 | A2001-7 | 1904 | 4802 | 4790 |
| DTH188 | A2001-8 | 709 | 5904 | 2955 |
| DTH189 | A2001-9 | 1752 | 4039 | 3911 |
| DTH190 | A2001-10 | 1146 | 4290 | 3811 |
| DTH191 | A2001-11 | 1242 | 4714 | 4343 |
| DTH192 | A2001-12 | 2282 | 4281 | 4573 |
| DTH193 | A2001-13 | 2340 | 5203 | 4522 |
| DTH194 | A2001-14 | 2579 | 4334 | 5016 |
| COMH195 | COMH195 | 917 | 5504 | 4364 |
