# Supplementary material for: Approaches and progress in breeding drought‐tolerant maize hybrids for tropical lowlands in west and central Africa
Source: Plant Genome. 2024 Feb 20;17(2):e20437. doi: 10.1002/tpg2.20437 (PMC12806867; doi:10.1002/tpg2.20437)
Supplement: Supplementary file 3 [file TPG2-17-e20437-s003.docx]

**Materials and methods**

**Genetic Materials**

The collaborative regional trials targeted for genetic gain analyses consisted of 148 drought tolerant hybrids (DTH), 41 commercial hybrids (COMH), and farmers-preferred varieties (FLV) (Supplementary Table S2) conducted for 11 years (2011-2021). The DTH hybrids were formed from parental lines developed through repeated inbreeding and screening under managed drought stress conditions at Ikenne in Nigeria. The commercial hybrids originated from national, regional, and multi-national seed companies and marketed in many African countries. The local maize variety (LOCAL) was a farmer preferred recycled hybrid or an improved open-pollinated maize variety commonly grown around the testing site where the collaborative regional trials were conducted. In these regional trials, 10 to 21 new hybrids were constantly added, while the least productive hybrids were removed. We kept one DTH and two COMH as common checks for 11 years to maintain the connectivity of the data. Furthermore, 82 DTH and 24 COMH hybrids were advanced and re-evaluated for two to ten years, making the data sets well connected to the preceding testing years. The good connectivity of these data sets had thus allowed dependable genetic gain estimates in the present review.

**Evaluating regional trials under Manged Drought Stress and Full Irrigation**

The hybrids in each regional trial were sown in alpha lattice designs with two replications under managed drought stress (MDS) and full irrigation (WW) at the IITA experiment station in Ikenne (6^0^53’ N, 3^0^42’ E, altitude of 60 m) in Nigeria during the 2011/2012 to 2018/2021 dry seasons. Drought stress was induced in a block by withdrawing irrigation from 35 days after planting to harvesting time of the trials, whereas an adjacent block received full irrigation every week from planting until the hybrids attained physiological maturity. The withdrawal of water from 15 to 20 days before anthesis provides desirable stress levels at anthesis, silking and grain filling stages of maize (Bänziger et al., 2000). Each hybrid was planted in two rows of 4 m length with a spacing of 0.75 m between rows and 0.25 m spacing between plants within a row. In each hill, two seeds were planted with one plant left after emergence to attain a population density of 53,333 plants per ha. We applied a compound fertilizer at the rate of 60 kg N, 60 kg P and 60 kg K ha^-1^ at sowing and an additional 60 kg N ha^-1^ fertilizer was applied four weeks later. Pre-emergence herbicides were sprayed at the rate of 5-liter ha^-1^ that was followed by manual weeding to keep the trials weed-free.

**Evaluating regional trials in multiple rainfed environments (MET)**

The national and private sector partners planted hybrids included in regional trials in alpha lattice designs with three replications during the main rainy seasons in 249 test environments representing the diverse maize growing conditions in West Africa (Supplementary Figure S2). Each hybrid was planted in two rows of 5 m length with spacing of 0.75 m between rows and 0.5 m between plants within a row. The national and private sector partners used the recommended crop management practices when conducting the regional trials at their testing sites.

**Trait measurements**

The traits measured in each plot under MDS and WW conditions include days to anthesis and silking, anthesis-silking interval, plant and ear heights, number of ears per plant and grain yield. Days to anthesis and silking represent the number of days from planting to when 50% of the plants were shedding pollen and showed emerged silks, respectively. Anthesis-silking interval (ASI) was calculated as interval in days between dates of silking and anthesis. The distance from the base of the plant to the height of the first tassel branch and the node bearing upper ear were measured to obtain plant and ear heights, respectively. Husk cover was rated on a 1 to 5 scale, where 1= husks tightly arranged and extended beyond the ear tip and 5 = ear tips exposed. Ear aspect was scored on a 1 to 5 scale, where 1 = uniform, large, and well-filled ears and 5 = variable, small and partially filled ears. All ears harvested from each plot were shelled and representative kernel samples were taken to measure moisture that was used to compute grain yield adjusted to 15% moisture. The national and private sector partners recorded the same sets of traits described above in the regional trials conducted in multiple test environment for 11 years. In the collaborative trials, grain yield was calculated using ear weight assuming a shelling percentage of 80% and final adjusted moisture content of 15% in each testing site.

**Genetic gain estimates**

Analysis was also conducted for each trait recorded across MET using the model provided by Mackay et al. (2011). In this analysis, the year and location combinations were treated as environments.

where y_irbjk_ is the yield of the ith hybrid of the bth block nested into the rth replication in the jth location and kth year; μ is the overall mean; G_i_ is the effect of the ith hybrid; Y_k_ is the effect of the kth year; R_rjk_ is the effect of the rth replication in the jth location and kth year; B_brjk_ is the effect of the bth block nested into the rth replication in the jth location and kth year; GY_ik_ is the ith hybrid year interaction effect; S_jk_ is the effect of location j within year k; e_ijk_ is the residual, attributable to the combined effects of within-trial error and hybrid location within-year interaction. All effects except μ, G_i_ and Y_k_ were assumed to be random (Mackay et al., 2011), assuming heterogeneous residual variances to fit separate residual variance for each year.

The analysis was conducted for each trait measured under MDS and WW conditions at Ikenne using the model provided by Mackay et al. (2011) as above, where the component location was dropped.

We adopted a two-stage analysis in which hybrids and years were first treated as fixed factors in a combined analysis within each environment for MET and eah year for MDS and WW. Analyses of individual trials had been performed to estimate repeatability values for each year under MDS and WW conditions and for each location x year combination across MET using a mixed model with the hybrids as random effects as suggested by Cullis et al. (2006). Trials conducted under MDS and WW conditions, and in each METs with repeatability values of less than 0.2 were discarded. Trends were then determined in a second stage of analysis by a weighted regression of the estimated hybrids adjusted means on year of origin within each environment or year. The weights were the inverse of the squared standard errors of the adjusted means of the hybrids. The percent change in genetic gain for MDS, WW, and MET attributable to genetic causes was estimated as a ratio of the regression slope to the y-intercept of the regression plus the slope multiplied by the year of first testing.

**Genetic correlations**

To understand heritable relationships among environments and to determine whether these relationships change across testing conditions, genetic correlations were computed for each pair of growing conditions following a two-stage method of analysis proposed by Smith et al.(2001). In the first stage, we conducted single-trial analyses to estimate the Best Linear Unbiased Estimators (BLUEs) and their corresponding weights, which are the diagonal of the inverse of the variance-covariance matrix of the predictions’ errors. In the second stage, we performed a weighted linear mixed model analysis for the combined model. We modeled the Genotype-by-Environment (GxE) as nested effects considering a general heterogeneous correlation structure. This process was carried out for yield.

Cullis, B. R., Smith, A., and Coombes, N. (2006). On the design of early generation variety trials with correlated data. *J. Agric. Biol. Environ. Stat.* 11, 381–393

Mackay, I., and Horwell, A. (2011). Garner, J. et al. Reanalyses of the historical series of UK variety trials to quantify the contributions of genetic and environmental factors to trends and variability in yield over time. *Theor. Appl. Genet.* 122, 225–238 <https://doi.org/10.1007/s00122-010-1438-y>

Smith, A. B., Cullis, B. R., and Gilmour, A. (2001). The analysis of crop variety evaluation data in Australia. *Aust N. Z. J Stat* 43, 129–145
